# Overcoming Challenges in Retrieving an Encapsulated Rear Tip Extensor During Conversion From Malleable to Inflatable Penile Prosthesis Surgery: A Case Report and Review of Literature

**DOI:** 10.7759/cureus.75239

**Published:** 2024-12-06

**Authors:** Laura Angulo-Llanos, Armin Ghomeshi, Thomas A Masterson

**Affiliations:** 1 Department of Urology, University of Miami Miller School of Medicine, Miami, USA; 2 Department of Urology, Florida International University Herbert Wertheim College of Medicine, Miami, USA

**Keywords:** cavernoscopy, corporotomy, inflatable penile prosthesis, malleable penile prosthesis, perineal incision, rear tip extender

## Abstract

Insertion of inflatable penile prosthesis (IPP) is generally regarded as a safe procedure, with low rates of complications. However, when complications do arise, they can pose significant challenges to both patients and surgeons. Patient optimization and adherence to specific intraoperative protocols are crucial in mitigating the risk of surgical complications. Standardization of intraoperative management, including the administration of intravenous antibiotics, meticulous aseptic techniques, and antibiotic irrigation of the prosthesis, is paramount. Furthermore, having the appropriate set of instruments can reduce operative time and minimize tissue manipulation. While the literature predominantly focuses on outcomes such as infection, prosthesis malfunction, corporal perforation, and penile length loss, reports on managing retained prosthetic components are rare. This uncommon complication raises important considerations regarding whether foreign bodies should be left in place or removed during revision penile prosthetic surgeries. We present the case of a 49-year-old male who underwent IPP insertion. Following complications due to infection, salvage penile prosthetic surgery was performed 38 days after penile prosthesis insertion, using a temporary malleable implant. Subsequently, eight months later, the patient underwent surgery for the replacement of the malleable implant with an IPP. During the replacement procedure, the right-side malleable component was removed easily, while the left-side component was discovered to have the rear tip missing. Removal attempts using intracorporeal rigid cystoscopy and graspers were unsuccessful, necessitating a perineal counter incision for retrieval. After successfully removing the encapsulated extensor, a new IPP was successfully placed without complications.

## Introduction

Penile prosthesis surgery is a common and safe treatment option for erectile dysfunction unresponsive to medical therapy. Penile prostheses are categorized into two groups: inflatable and non-inflatable. Despite its safe profile, complications such as infection and mechanical issues can arise, necessitating revision surgeries [1-3].

Most reported cases concerning post-implantation complications predominantly focus on mechanical failure and infection. Salvage procedures are often performed to replace an infected inflatable penile prosthesis (IPP) with a temporary malleable device after an extensive washout, which can prevent penile loss and maintain the corporeal spaces for future re-implantation of a new inflatable device. This scenario is particularly common among patients with diabetes mellitus, as they are at higher risk of implant infections [4].

It is worth noting that there is limited literature addressing encapsulated foreign bodies from salvage procedures, and it remains a subject of debate whether they should be or not be removed. We present a case of encapsulated rear tip extensor retention during conversion from a malleable penile prosthesis to an IPP, highlighting the challenges encountered and the successful resolution.

## Case presentation

A 49-year-old male who underwent the initial insertion of an IPP presented to the emergency department approximately eight weeks post surgery complaining of testicular pain, fever, and swelling with drainage from the scrotum. Physical examination revealed diffuse scrotal edema, serous drainage, and erythema, suggesting an abscess. His past medical history included hypertension, diabetes mellitus (DM), erectile dysfunction, and bladder cancer (diagnosed seven years earlier). At the time of the presentation, he was taking lisinopril, hydrochlorothiazide, metformin, and rosuvastatin.

**Figure 1:**
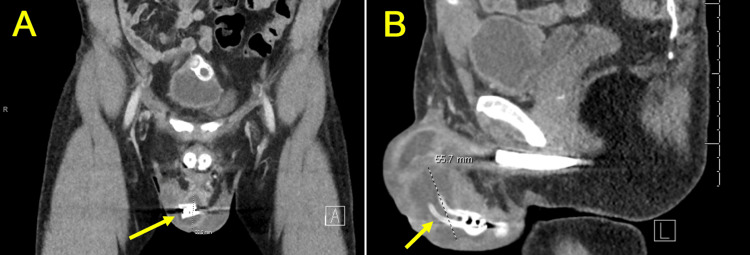
Pelvic CT scan with contrast (A) Coronal view showing an enhanced amorphous hypodensity surrounding the penile implant reservoir, potentially indicating an abscess (arrow) and (B) Sagittal view highlighting a 5.6 cm hypodense area in the mid-scrotum adjacent to the penile implant, with implant tubing passing through it (arrow).

A CT scan of the pelvis with contrast confirmed the presence of a 5.6 cm abscess adjacent to the penile implant reservoir (Figure 1). The patient was started on vancomycin, levofloxacin, and cefepime, and underwent salvage surgery for removal of the IPP. A temporary malleable implant was inserted to prevent penile length loss, facilitate infection control, and allow tissue healing before IPP reimplantation, as immediate replacement with an IPP carries a higher risk of reinfection. The surgery was successful, and the patient recovered well without any postoperative reinfection.

Subsequently, eight months later, the patient underwent surgery for the replacement of the malleable implant with an IPP. During the replacement procedure, the right-side malleable component was easily removed in total, while the left-side component was found to have the rear tip missing. Brooks dilators were inserted into the corpora, revealing a clear difference in corporal depth, suggesting that the rear tip was still inside the left corpora. The initial assessment involved the insertion of a rigid cystoscope into the left corpora, which confirmed the presence of the rear tip partially encapsulated in a pseudo capsule, suggesting tissue ingrown between the cylinder and rear tip during the healing process, resulting in detachment. Initial attempts to grab the rear tip with rigid graspers were unsuccessful. Subsequently, the cystoscope was retracted, and Metzenbaum scissors were employed to incise the scar tissue holding the rear tip in the corpora. Multiple cavernoscopy attempts were made to grasp the rear tip using both flexible and rigid graspers without success. 

**Figure 2:**
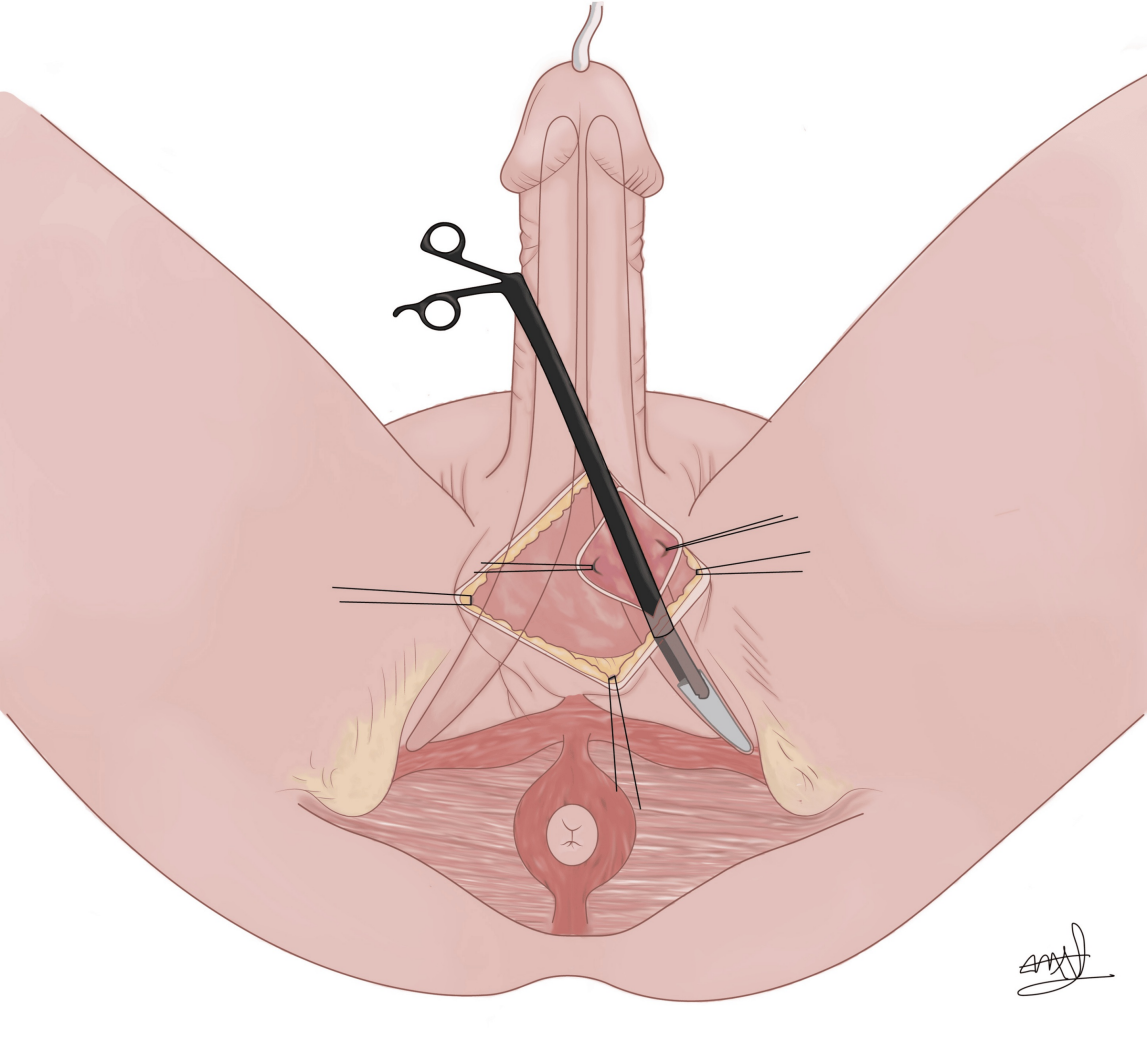
Perineal incision and right corporotomy to retrieve the rear tip. Image Credit: Maria Andrea Angulo Llanos (mariandreangulo99@gmail.com). All rights reserved ©2024.

We then decided to remove the rear tip through a separate perineal incision. A dilator was introduced into the corpora to demarcate a perineal incision, followed by dissection towards the dilator to access the corpora. Subsequent corporotomy facilitated the successful identification and extraction of the rear tip (Figure 2). The proximal corpora was closed over the dilator tip with a 2-0 PDS suture.

After retrieval, irrigation with Irrisept (chlorhexidine gluconate) was performed to ensure thorough cleansing before closing the incision. Then, a solution with 50% diluted 3% hydrogen peroxide was applied over the entire operative field to ensure sterility and mitigate the risk of infection. Fresh drapes were applied, and gloves and gown were changed. Upon re-draping, additional irrigation with chlorhexidine was administered. Following a thorough washout, the corporal spaces were meticulously measured, revealing symmetrical dimensions without evidence of perforation or crossover. We decided to perform a wind sock on the left corpora to prevent proximal migration postoperatively. Following more antibiotic washout, a new IPP was implanted without further complications.

The patient's recovery was uneventful. He was discharged the next day and followed up regularly without signs of infection. At the six-week follow-up, the implanted IPP functioned effectively, restoring erectile function in the patient.

## Discussion

Revision penile prosthesis surgery following device infection is challenging, with significantly higher infection rates and increased procedural complexity compared to primary implantation due to the presence of corporal fibrosis and hypoplastic scrotal or penile skin [2,5]. 

During the healing phase of salvage procedures, the formation of a pseudo capsule between the malleable cylinder and the rear tip extensor can occur, leading to detachment [6,7]. Because of its rarity, there is limited literature addressing this phenomenon, emphasizing its status as a challenging complication in penile prosthesis surgery. In this case, a conventional cavernoscopy using a rigid cystoscope and graspers was ineffective, necessitating the adoption of an alternative approach. A corporotomy via perineal incision allowed for safe and effective removal of the encapsulated extensor. 

Prior to salvage procedures, preoperative physical examination and CT scan of the abdomen and pelvis should be conducted to identify implant components [5,8]. However, in this instance, neither the physical examination nor imaging studies identified the detached rear tip; instead, it was solely a surgical finding.

Kava and Burdick-Will presented a series of four patients with complications resulting from failure to remove all foreign bodies associated with IPPs [5]. They also found that intraoperative cultures for the four patients were positive for *Staphylococcus epidermidis*, a skin commensal that thrives on prosthetic surfaces, forming biofilms that are difficult to eradicate. This bacterium is particularly adept at colonizing foreign materials, such as implants, and can lead to persistent infections if not fully cleared. Failure to remove all foreign bodies from the operative field may result in chronic infection, often necessitating further surgical intervention [9]. Given the diabetic status of the patient in the current report as a primary risk factor, infection could potentially result in more aggressive tissue damage and septicemia. 

This raises a debate over whether foreign bodies should be left in place or removed during revision penile prosthetic surgeries. Studies suggest that removing all foreign materials is crucial for reducing the risk of persistent infection. However, there are concerns that using more invasive techniques for removal may increase the risk of infection and other post-surgical complications. Some advocate leaving foreign bodies in situ in a non-infected revision case. This has become a standard practice for difficult-to-retrieve reservoirs; the “drain and retain” technique [10]. However, retaining foreign materials, on the other hand, can lead to chronic infections, particularly with organisms like *S. epidermidis*, which form biofilms on prosthetic surfaces. While retaining these materials might reduce immediate surgical trauma, the long-term risks, such as persistent infections and potential legal issues, may outweigh the short-term benefits. The current literature on this subject is limited, highlighting the need for further research to determine whether the risks of leaving foreign materials in place are greater than those posed by their removal. 

We advocate for the removal of foreign materials rather than leaving them in place. To further reduce risks and support optimal recovery, we recommend that patients stay in the hospital for at least one night for close monitoring after the procedure. This approach allows for the administration of intravenous antibiotics, tailored to the specific pathogens identified, and ensures vigilant oversight of the patient’s recovery.

Additionally, we emphasize the importance of a comprehensive healing phase post surgery. During this period, it is essential to monitor for signs of infection, assess healing progress, and address any complication that may arise. By implementing these measures, we aim to improve patient outcomes and reduce the likelihood of adverse effects associated with foreign material retention. This protocol not only supports immediate postoperative care but also contributes to long-term success in revision penile prosthetic surgeries. 

Individual variability in patient anatomy and clinical presentation could lead to different outcomes in similar scenarios. Furthermore, the follow-up period of one year, while sufficient to evaluate short-term success and the absence of reinfection, does not provide insight into long-term outcomes such as device durability and patient satisfaction.

## Conclusions

This case underscores the complexity of penile prosthesis revision surgeries, particularly in cases involving encapsulated foreign bodies such as the rear tip extender. Despite the inherent challenges, including failed initial extraction attempts, our approach using a secondary perineal incision allowed for the successful removal of the encapsulated rear tip, followed by the safe implantation of a new IPP. The patient's uneventful recovery and restoration of erectile function further highlight the importance of meticulous surgical planning and rigorous infection control measures in minimizing postoperative complications.

Additionally, this case also raises awareness of the potential for tissue ingrowth leading to encapsulation of prosthetic components, an issue not widely discussed in current literature. Future studies are warranted to establish whether the removal of encapsulated foreign bodies in similar scenarios is necessary to prevent future complications or whether observation may suffice. As seen in this case, timely intervention and individualized treatment can lead to successful outcomes, even in complex revision surgeries for penile prostheses.
